# Association analysis of single nucleotide polymorphisms in the sheep *FecB* gene with reproductive and body size performance

**DOI:** 10.5194/aab-67-81-2024

**Published:** 2024-02-15

**Authors:** Lingchao Kong, Shuaitong Li, Yuan Pan, Jiaqi Li, Siyi Li, Yining Liu, Sibing Hou, Qingkun Liu, Yanjun Qiao, Yinggang Sun, Zeying Wang

**Affiliations:** 1 College of Animal Science and Veterinary Medicine, Shenyang Agricultural University, Shenyang 110866, China

## Abstract

The *FecB* gene is one of those responsible for regulating reproductive traits in sheep. This experiment was designed to explore the impact of SNPs (single nucleotide polymorphisms) within the *FecB* gene on both the reproductive and body size performance of sheep. In this experiment, two SNP sites were identified as C413217T and A431965G. Through analysis of genetic diversity and correlations, we aimed to identify combinations of genotypes and haplotypes that influence reproductive performance and body size performance. The most prominent genotypes associated with lambing performance were identified as AA and CT, with the dominant haplotype combination being AACT. For body size performance, the dominant genotypes were AA and CC, while the dominant haplotype combination was AACC. These advantageous genotypes and haplotype combinations are conducive to promoting the selection and improvement of sheep breeds, advancing the progress of sheep genetic breeding, and providing theoretical support for the breeding of higher-fertility sheep.

## 1 Introduction

China boasts a rich heritage of sheep farming, with one of the world's most extensive and diverse populations of sheep breeds (Xu et al., 2020). Sheep are one of the most important economic animals in China. Its reproductive traits are one of the indicators of its economic traits (Yang et al., 2023). Sheep exhibit seasonal estrous behavior, often resulting in relatively low lambing rates. In order to maximize economic benefits, researchers have begun to study how to improve the lambing rate of ewes. In recent years, various artificial interventions have been employed to augment the reproductive capacity of sheep, i.e., simultaneous estrus treatment, artificial insemination (AI), and multiple ovulation and embryo transfer (MOET) (Yang et al., 2023). Furthermore, researchers have identified specific genes associated with multiple births in certain sheep breeds. As of 2022, a total of 16 genes linked to multiple births have been identified (Nan et al., 2022). Each of these genes wields a substantial influence on the reproductive performance of sheep, offering promising avenues for advancing the efficiency of sheep-breeding practices.

The Booroola fecundity gene (*FecB*), a mutant of bone morphogenetic protein 1B (*BMPR-1B*) that was discovered in Booroola Merino, was the first prolificacy gene identified in sheep related to increased ovulation rate and litter size (Xie et al., 2023). The mutation is an A746G mutation in the coding region of the gene that results in the substitution of glutamine for arginine at position 249 in the protein sequence (Q249R) (Gong et al., 2023), altering the kinase structural domain of the *BMPR1B* gene (Akhatayeva et al., 2023). The effects of the *FecB* gene on sheep reproductive performance are notable. Its impact is additive on the number of ovulations and is partially dominant in terms of lambing (Liu et al., 2014). Compared to the wild-type ( ${}^{++}$ ) individuals, possessing a single copy of the *FecB* gene increased the number of ovulations and lambs by 1.65 and 0.67, respectively. Having two copies of the gene resulted in even greater increases, with 3.61 additional ovulations and 0.77 more lambs (Guo, 2018). It is worth mentioning that the effects of the *FecB* gene on the number of ovulations and lambing can vary among different sheep breeds. For instance, when compared to the wild type ( ${}^{++}$ ), the average number of lambs produced increased by 0.16 for individual Tan sheep type BB and 1.89 for Small-tailed Han Sheep type BB (Guo, 2018). The impact of the *FecB* gene mutation on sheep reproductive performance has garnered significant attention from researchers and scholars worldwide. They have delved into the diverse effects of *FecB* gene polymorphisms on the growth and development of various sheep breeds, with findings reported both domestically and internationally. One noteworthy study conducted by Gootwine et al. (2006) focused on sheep carrying the *FecB* (Booroola) mutation. The results of this investigation revealed that lambs harboring the *FecB* gene exhibited lower birth weights, slower rates of growth after weaning, and lighter mature body weights in ewes. In essence, lambs with the *FecB* gene demonstrated a distinct growth pattern characterized by reduced birth weights and slower post-weaning growth, which subsequently led to lighter mature weights in ewes. Similarly, overseas researchers have also shed light on the effects of the *FecB* gene. Their studies indicate that ewes carrying the *FecB* gene tend to exhibit smaller body size traits when compared to those not carrying the gene (Sejian et al., 2015). This observation further underscores the multifaceted influence of the *FecB* gene on sheep development, particularly with regard to body size characteristics. The collective body of research on the *FecB* gene underscores its significance in shaping various aspects of sheep physiology and performance, offering valuable insights for breeding programs and agricultural practices.

Single nucleotide polymorphisms (SNPs) were proposed by Lander (1996). SNP belongs to a kind of genetic marker. It has many advantages, such as a high number and wide distribution, high genetic stability, and easy automation. Common SNPs typically manifest as bi-allelic polymorphisms, and they can be found in both the coding and non-coding regions of a gene. When SNPs occur in the coding region, they can profoundly influence the function of the gene, consequently leading to alterations in various biological traits. As a result, the study of SNPs holds immense significance in the realm of animal genetics. Nowadays many scholars have found other SNP sites in the *BMPR1B* gene besides the *FecB* mutation. For instance, Souza et al. (2001) uncovered both the *FecB* mutation site and the C1113A mutation site. Chu et al. (2011) detected 20 fresh mutation sites within the gene. Notably, three of these sites – G922T, T1043C, and G192A – led to alterations in amino acids. Remarkably, despite these changes, they exhibited no substantial impact on lambing numbers. Gao et al. (2021) sequenced all exonic regions of the *FecB* gene in Mongolian sheep breeds and detected 10 novel variants by direct sequencing. Among them, two SNP sites, C29346567T and G1470T, significantly increased litter size.

The aim of this experiment was to investigate the effect of SNPs in the *FecB* gene of sheep on their reproductive performance and body size performance. By conducting an analysis of SNP genetic diversity and investigating the association between reproductive performance and body size performance, we aimed to identify specific genotypes and haplotype combinations that exerted an influence on these aspects. This research has significant implications for the enhancement and selection of sheep breeds. It contributes to the advancement of genetic breeding practices in the sheep industry and offers valuable theoretical support for the development of sheep with heightened fertility levels.

## 2 Materials and methods

### 2.1 Experimental animals and body size performance data and reproductive performance data

This study involved a sample of 1137 ewes from various breeds, collected at Tianfeng Breeding Farm in Zhangwu County, Fuxin, Liaoning Province, China. They consisted of 777 Charolais ewes, 120 Australian White ewes, 120 Charolais × Small-tailed Han Sheep crossbred generation ewes (CS), and 120 Australian White × Small-tailed Han Sheep crossbred generation ewes (AS). All the selected ewes were healthy 3-year olds, and they were bred to a consistent standard. Special care was taken to minimize any discomfort experienced by the ewes during blood sample collection. For DNA extraction, blood samples were obtained from the jugular vein of each sheep, with guidance from a qualified veterinarian. Additionally, data pertaining to body size and lambing numbers were provided by Tianfeng Breeding Farm. The body size data encompassed various measurements, including weight, body height, back height, waist height, sacral height, hip height, frontal width, tube circumference, chest circumference, limb length, leg length, straight body length, chest depth, chest width, waist angle width, and hip width. This comprehensive dataset served as the basis for the study's analysis and findings.

### 2.2 DNA extraction and primer design

DNA was extracted from 1 mL of blood sample per sheep using the Ezup Column Blood Genomic DNA Purification Kit (Shanghai Bioengineering Co. Ltd, shanghai, People's Republic of China). Extracted DNA is stored at - 20 ${}^{\circ }$ C. The *FecB* gene reference sequence was obtained from the National Center for Biotechnology Information (NCBI) database (Reference Sequence NC_056059.1) and specific primers were designed by Premier5.0. The primers were synthesized by Shanghai Biotechnology Co. Ltd. (Table 1).

**Table 1:** Amplification and typing primers for the *FecB* gene target fragment.

Gene	Sense primer	Anti-sense primer	Temperature	Product	Regions
	(forward)	(reverse)	( ${}^{\circ }$ C)	(bp)	
*FecB*	5 ${}^{\prime }$ CACACACTTACTTTGCC3 ${}^{\prime }$	5 ${}^{\prime }$ TACATTCATTCCGTTCT3 ${}^{\prime }$	52.0/52.0	707	412 810–413 517
(C413217T)					
*FecB*	5 ${}^{\prime }$ TCAGCACTCAAAACCAACAG3 ${}^{\prime }$	5 ${}^{\prime }$ ATTTCAAGTCCACCATCCAT3 ${}^{\prime }$	52.9/50.9	786	431 431–432 217
(A431965G)					

### 2.3 PCR (polymerase chain reaction) reaction system

In this study, the PCR reaction system had a total volume of 50 µ L composed of the following components: 25 µ L of 2 × SanTaq PCR Mix solution, 1 µ L of DNA template, 2 µ L of upstream primer, 2 µ L of downstream primer, and 20 µ L of ddH ${}_{2}$ O (deionized water). These reagents were meticulously added to a PCR tube, thoroughly mixed with agitation and subsequently centrifuged to ensure uniform distribution. The PCR reaction followed a specific set of conditions as outlined below: initial denaturation at 94 ${}^{\circ }$ C for 5 min, a denaturation step at 94 ${}^{\circ }$ C for 30 s, an annealing step with the temperature adjusted to 48 ${}^{\circ }$ C for 30 s, an extension at 72 ${}^{\circ }$ C for 30 s, and a final extension step at 72 ${}^{\circ }$ C for 10 min.

**Figure 1:**
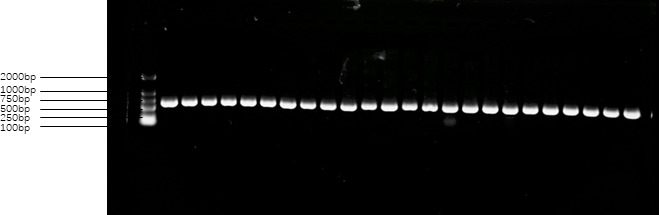
Amplification products of primer 1 of the *FecB* gene. Note: the first band from left to right is the MARKER, the second to seventh bands are Charolais sheep, the eighth to 13th bands are Australian White sheep, the 14th to 19th bands are CS, and the 20th to 24th bands are AS. DNA templates were randomly selected in all the samples.

**Figure 2:**
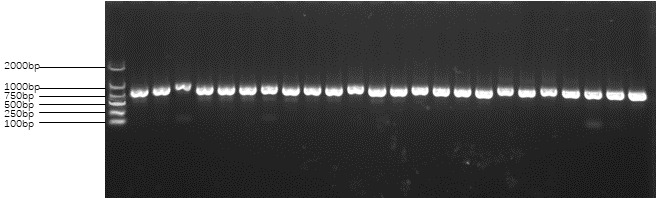
Amplification products of primer 2 of the *FecB* gene. Note: the first band from left to right is the MARKER, the second to seventh bands are Charolais sheep, the eighth to 13th bands are Australian White sheep, the 14th to 19th bands are CS, and the 20th to 24th bands are AS. DNA templates were randomly selected in all the samples.

Following the PCR reaction, electrophoresis was conducted at 130 V with a power setting of 180 W for a duration of 20 min. After electrophoresis, the presence or absence of the target DNA fragment was observed within the electrophoresis band. If the target fragment was detected, the sample was subsequently sent to Shanghai Biotechnology Co. Ltd., as depicted in Figs. 1 and 2.

### 2.4 Statistical analysis

In this study, various genetic parameters and statistical analyses were performed to assess the genetic diversity and its impact on performance traits in multi-breed sheep. The major ones included genotype and allele frequencies, polymorphism information content (PIC), number of effective alleles (Ne), and heterozygosity (He). To investigate the relationship between the *FecB* gene and body size performance traits in multi-breed sheep, a one-way analysis of variance (ANOVA) was conducted using SPSS software (Park et al., 2009). The completeness of the animal model was analyzed using the following equation:

1 Yijkl=μ+hi+pj+sk+mL+eijkl.

Yijkl is the observed value, μ is the overall average, hi is the influence of genotype or combined haploid, pj is the influence of season and farm, sk is the influence of year, mL is the influence of paternal decline, and eijkl is the random error. The significance of the results was assessed based on p values. The results should be presented in the format of “mean ± standard error”, which provides both the central tendency (mean) and the variability (standard error) of the data.

## 3 Results

### 3.1 SNP site sequencing maps

**Figure 3:**
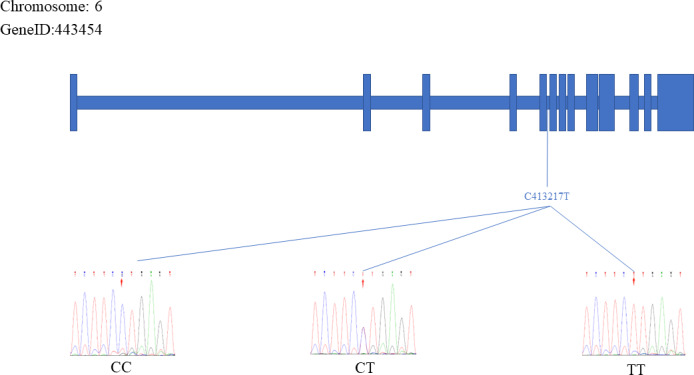
The C413217T site of the *FecB* gene.

**Figure 4:**
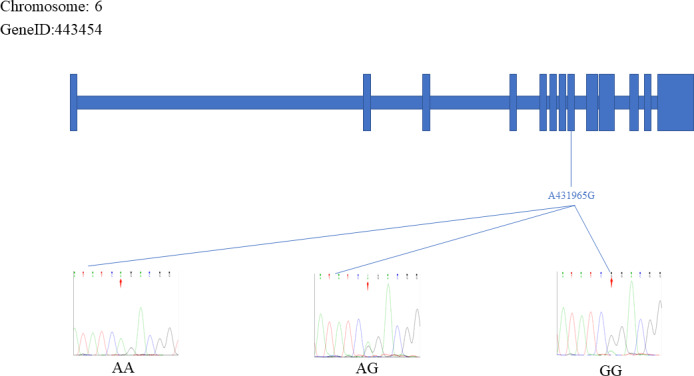
The A431965G site of the *FecB* gene.

The sequencing results were aligned and compared with the reference *FecB* gene sequence, which resulted in the identification of two specific SNP mutation sites within the *FecB* gene: C413217T and A431965G. Figures 3 and 4 depict the positions of the two SNP sites. The figure represents the exonic region of the *FecB* gene on Chr6.

### 3.2 Population genetic analysis

The genotype frequencies and allele frequencies of the two SNP mutation sites of the *FecB* gene in each breed of sheep are shown in Table 2. In Charolais × Small-tailed Han Sheep crossbred generation ewes and Australian White × Small-tailed Han Sheep crossbred generation ewes, the polymorphic information content (PIC) at the C413217T site is less than 0.25, indicating a low level of polymorphism. In Charolais ewes, the PIC is greater than 0.25 but less than 0.5, suggesting medium polymorphism. In Australian White ewes and Charolais ewes, the PIC of A431965G is less than 0.25, indicating low polymorphism. In Charolais × Small-tailed Han Sheep crossbred generation ewes and Australian White × Small-tailed Han Sheep crossbred generation ewes, the PIC at this site is greater than 0.25 but less than 0.5, indicating medium polymorphism. Of the two SNP sites, the heterozygosity (He) and the number of effective alleles (Ne) were higher at the A431965G site, suggesting a greater degree of genetic variation at this site. At the C413217T site, the χ 2 value is greater than 1, and the p value is less than 0.05 in Charolais ewes, Charolais × Small-tailed Han Sheep crossbred generation ewes, and Australian White × Small-tailed Han Sheep crossbred generation ewes. This indicates that these populations are not in Hardy–Weinberg equilibrium. This suggests that there is significant deviation from the expected genetic frequencies, possibly due to strong artificial selection practices, which can affect gene and genotype frequencies.

**Table 2:** Population genetic analysis of sites C413217T and A431965G in various breeds of sheep.

Name	Site	Specimens	Genotype frequency			Allelic frequencies		PIC	He	Ne	$\chi^{2}$	P
			MM	Mm	mm	M	m					
CS	C413217T	120	0.70	0.30	0	0.85	0.15	0.22	0.26	1.34	0.93	0.33
AS	C413217T	120	0.97	0.03	0	0.98	0.02	0.03	0.03	1.03	0.01	0.93
Australian White ewes	C413217T	120	1	0	0	1	0	0	0	1		
Charolais ewes	C413217T	777	0.66	0.30	0.04	0.81	0.19	0.26	0.30	1.44	$3.45722\times 10^{-5}$	0.99
SC	A431965G	120	0.6	0.17	0.23	0.68	0.32	0.34	0.43	1.76	11.34	0.01
AS	A431965G	120	0.54	0.13	0.33	0.6	0.4	0.36	0.48	1.92	15.65	$7.6287\times 10^{-5}$
Australian White ewes	A431965G	120	0.87	0.13	0	0.93	0.07	0.12	0.12	1.14	0.15	0.70
Charolais ewes	A431965G	777	0.98	0	0.02	0.98	0.02	0.03	0.03	1.04	171	$4.4747\times 10^{-39}$

### 3.3 Analysis of gene substitution effects

The additive effect values of the C413217T and A431965G sites within the *FecB* gene were found to be negative across various sheep breeds, including Charolais ewes, Australian White ewes, Charolais × Small-tailed Han Sheep crossbred generation ewes, and Australian White × Small-tailed Han Sheep crossbred generation ewes. These negative values indicate that mutations at these specific sites have a positive impact on enhancing production performance in all cases. Notably, Charolais ewes exhibited the most pronounced improvements in production performance attributable to these mutations. Specifically, the replacement of C with T at the C413217T site led to a substantial 56.06 % enhancement, while the substitution of A with G at the A431965G site resulted in an even more remarkable 111.72 % enhancement (Table 3).

**Table 3:** Analysis of gene replacement effects at sites C413217T and A431965G.

Name	Site	Dominant	Additive	Average effect	Average effect	The average effect
		effect	effect	of the u gene	of the U gene	of u instead of U
		d	a	a1	a2	a
CS	C413217T	- 1.5	- 10.5	- 7.39	3.62	- 11.01
AS	C413217T	- 13.5	- 14.5	- 12.82	6.29	- 19.11
Australian White ewes	C413217T	- 15	- 15	- 13.50	6.62	- 20.12
Charolais ewes	C413217T	- 7.5	- 53.5	- 37.61	18.45	- 56.06
CS	A431965G	- 7.5	- 5.5	- 5.41	2.65	- 8.06
AS	A431965G	- 9	- 3	- 4.08	1.99	- 6.08
Australian White ewes	A431965G	- 9	- 13	- 10.79	5.29	- 16.08
Charolais ewes	A431965G	- 85.5	- 82.5	- 74.96	36.77	- 111.72

### 3.4 Analysis of the relationship between SNPs and reproductive performance

From Table 4, The CT genotype at the C413217T site demonstrates superior reproductive performance compared to both the CC and TT genotypes. Specifically, the CT genotype appears to yield more favorable outcomes. At the A431965G site, the AA genotype showcases superior reproductive performance in comparison to the GG genotype. This suggests that the AA genotype may lead to more favorable reproductive outcomes. It is noteworthy that the CT genotype at the C413217T site appears to result in the highest number of lambs produced, indicating its potential significance in enhancing lamb production.

**Table 4:** Reproductive performance of the C413217T site and the A431965G site in various breeds of sheep.

Name	Site	Genotype	Number of lambs
Charolais ewes	C413217T	CC	1.74 ± 0.04 ${}^{\mathrm{aA}}$
	C413217T	CT	1.77 ± 0.07 ${}^{\mathrm{aA}}$
	C413217T	TT	1.00 ± 0.00 ${}^{\mathrm{bB}}$
	A431965G	AA	1.74 ± 0.35 ${}^{a}$
	A431965G	GG	1.00 ± 0.00 ${}^{b}$
Charolais × Small-tailed Han Sheep crossbred generation ewes	C413217T	CC	1.67 ± 0.05
	C413217T	CT	2.00 ± 0.00
	A431965G	AA	2.00 ± 0.00
	A431965G	AG	2.00 ± 0.00
	A431965G	GG	1.00 ± 0.00
Australian White × Small-tailed Han Sheep crossbred generation ewes	C413217T	CC	1.65 ± 0.04
	C413217T	CT	2.00 ± 0.00
	A431965G	AA	2.00 ± 0.00
	A431965G	AG	2.00 ± 0.00
	A431965G	GG	1.00 ± 0.00
Australian White ewes	C413217T	CC	2.00 ± 0.00
	A431965G	AA	2.00 ± 0.00
	A431965G	AG	2.00 ± 0.00

Note: different lower-cased letter superscripts indicate significant differences at the 0.05 level, while upper-cased letter superscripts indicate differences at the 0.01 level.

### 3.5 Analysis of the relationship between SNPs and body size performance

Table 5 presents the impact of two SNP mutation sites on body size performance in Charolais ewes. The AA genotype at the A431965G site demonstrates superiority over the GG genotype in terms of body weight, chest circumference, limb length, straight body length, chest width, and hip width. Additionally, the AA genotype is significantly superior to the GG genotype for tube circumference. However, for certain traits like body height and frontal width, the GG genotype surpasses the AA genotype. The CC genotype at the C413217T site exhibits superiority over other genotypes for weight, body height, and frontal width. The CC genotype is also significantly superior to the TT genotypes for tube circumference. In contrast, the TT genotype surpasses other genotypes for chest circumference, limb length, and leg length. Notably, the TT genotype is very significantly superior to the CT genotype for chest depth and chest width.

**Table 5:** Body size performance of the C413217T and A431965G sites in Charolais sheep.

Name	Charolais ewes				
Site	A431865G	A431865G	C413217T	C413217T	C413217T
Genotype	AA (765/777)	GG (12/777)	CC (509/777)	CT (239/777)	TT (29/777)
Weight (kg)	79.33 ± 0.70	70.83 ± 1.48	79.25 ± 0.78	79.14 ± 1.45	69.00 ± 0.00
Body height (cm)	71.59 ± 0.17	73.33 ± 1.24	71.81 ± 0.20 ${}^{a}$	71.24 ± 0.32 ${}^{a}$	68.00 ± 0.00 ${}^{b}$
Back height (cm)	71.21 ± 0.17	71.67 ± 1.24	71.37 ± 0.20	70.76 ± 0.30	70.00 ± 0.00
Waist height (cm)	71.73 ± 0.19	71.50 ± 0.98	71.77 ± 0.23	71.53 ± 0.28	72.00 ± 0.00
Sacral height (cm)	71.22 ± 0.20	72.67 ± 0.86	71.18 ± 0.27	71.43 ± 0.20	72.00 ± 0.00
Hip height (cm)	64.16 ± 0.31	68.33 ± 0.86	64.23 ± 0.38	64.31 ± 0.47	63.00 ± 0.00
Frontal width (cm)	15.50 ± 0.05	16.00 ± 0.21	15.60 ± 0.06	15.32 ± 0.08	15.00 ± 0.00
Tube circumference (cm)	9.74 ± 0.04 ${}^{a}$	8.50 ± 0.00 ${}^{b}$	9.84 ± 0.05 ${}^{a}$	9.40 ± 0.08 ${}^{\mathrm{ab}}$	9.00 ± 0.00 ${}^{b}$
Chest circumference (cm)	125.68 ± 0.54	122.67 ± 1.53	125.34 ± 0.63	126.05 ± 0.99	130.00 ± 0.00
Limb length (cm)	21.47 ± 0.13	23.00 ± 0.43	21.65 ± 0.16	20.98 ± 0.20	22.00 ± 0.00
Leg length (cm)	47.30 ± 0.38	40.83 ± 1.20	47.25 ± 0.42	46.00 ± 0.79	48.00 ± 0.00
Straight body length (cm)	64.10 ± 0.28	63.00 ± 1.54	64.32 ± 0.28	63.31 ± 0.68	67.00 ± 0.00
Chest depth (cm)	36.40 ± 0.14	36.67 ± 0.75	36.22 ± 0.17 ${}^{\mathrm{aA}}$	36.86 ± 0.20 ${}^{\mathrm{aA}}$	32.90 ± 0.00 ${}^{\mathrm{bB}}$
Chest width (cm)	34.88 ± 0.18	33.03 ± 0.49	34.42 ± 0.21 ${}^{\mathrm{aAB}}$	35.92 ± 0.29 ${}^{\mathrm{aA}}$	31.00 ± 0.00 ${}^{\mathrm{bB}}$
Waist angle width (cm)	32.00 ± 0.31	33.43 ± 0.80	31.42 ± 0.38	33.59 ± 0.49	33.20 ± 0.00
Hip width (cm)	34.40 ± 0.19	31.70 ± 1.10	34.26 ± 0.23	34.60 ± 0.36	31.00 ± 0.00

Note: different lower-cased letter superscripts indicate significant differences at the 0.05 level, while upper-cased letter superscripts indicate differences at the 0.01 level.

Table 6 presents the impact of two SNP mutation sites on body size performance in Charolais × Small-tailed Han Sheep crossbred generation ewes. At site A431965G, the AA genotype outperforms other genotypes across various body size metrics, including body weight, back height, sacral height, hip height, frontal width, tube circumference, chest circumference, limb length, straight body length, chest width, and hip width. Furthermore, the AA genotype exhibits significant superiority over the AG genotype in traits such as body height, back height, and waist angle width. Conversely, the GG genotype excels in leg length compared to the other genotypes. At C413217T, the CC genotype demonstrates superiority over the CT genotype for specific body size traits, including body weight, body height, back height, waist height, chest circumference, chest depth, chest width, waist angle width, and hip width. The CT genotype surpasses the CC genotype in other traits.

**Table 6:** Body size performance of the C413217T and A431965G sites in Charolais ×Small-tailed Han Sheep crossbred generation ewes.

Name	Charolais × Small-tailed Han Sheep crossbred generation ewes				
Site	A431965G	A431965G	A431965G	C413217T	C413217T
Genotype	AA (72/120)	AG (20/120)	GG (28/120)	CC (84/120)	CT (36/120)
Weight (kg)	69.53 ± 1.60	62.20 ± 0.24	62.86 ± 2.28	68.86 ± 1.39	61.83 ± 1.73
Body height (cm)	77.56 ± 0.57 ${}^{a}$	71.90 ± 0.40 ${}^{b}$	76.50 ± 0.69 ${}^{\mathrm{ab}}$	76.74 ± 0.51	75.50 ± 0.77
Back height (cm)	77.58 ± 0.55	73.70 ± 0.20	76.07 ± 0.64	76.98 ± 0.47	75.67 ± 0.68
Waist height (cm)	78.58 ± 0.56 ${}^{a}$	73.90 ± 0.18 ${}^{b}$	75.54 ± 0.59 ${}^{\mathrm{ab}}$	77.23 ± 0.51	76.78 ± 0.63
Sacral height (cm)	77.79 ± 0.51	75.00 ± 0.46	73.36 ± 1.31	76.25 ± 0.61	76.39 ± 0.68
Hip height (cm)	69.00 ± 0.53	67.00 ± 0.52	66.57 ± 1.36	68.05 ± 0.53	68.22 ± 0.92
Frontal width (cm)	15.58 ± 0.13	15.50 ± 0.23	15.00 ± 0.16	15.33 ± 0.12	15.67 ± 0.16
Tube circumference (cm)	10.00 ± 0.13	9.60 ± 0.37	9.64 ± 0.16	9.81 ± 0.13	9.94 ± 0.17
Chest circumference (cm)	128.44 ± 1.22	120.40 ± 1.43	122.21 ± 1.34	126.52 ± 1.11	123.61 ± 1.38
Limb length (cm)	22.14 ± 0.23	21.40 ± 0.23	21.57 ± 0.26	21.79 ± 0.16	22.11 ± 0.36
Leg length (cm)	52.06 ± 0.77	50.40 ± 0.94	54.64 ± 0.41	52.31 ± 0.57	52.26 ± 1.08
Straight body length (cm)	67.00 ± 0.77	63.00 ± 1.56	60.14 ± 0.65	63.79 ± 0.65	66.94 ± 1.26
Chest depth (cm)	39.46 ± 0.44	38.72 ± 0.65	39.50 ± 1.07	40.09 ± 0.47	37.62 ± 0.52
Chest width (cm)	34.51 ± 0.51	31.40 ± 1.10	31.79 ± 0.63	33.39 ± 0.53	33.28 ± 0.54
Waist angle width (cm)	28.85 ± 0.51 ${}^{a}$	23.76 ± 0.68 ${}^{b}$	27.74 ± 0.63 ${}^{\mathrm{ab}}$	28.19 ± 0.48	26.70 ± 0.65
Hip width (cm)	33.10 ± 0.72	32.56 ± 1.29	31.93 ± 0.89	33.20 ± 0.63	31.64 ± 0.92

Note: different lower-cased letter superscripts indicate significant differences at the 0.05 level, while upper-cased letter superscripts indicate differences at the 0.01 level.

Table 7 presents insights into the impact of two SNP mutation sites on body size performance in Australian White × Small-tailed Han Sheep crossbred generation ewes. At site A431965G, the AA genotype demonstrates superiority over the GG genotype in specific traits, including weight, leg length, straight body length, and frontal width. Notably, the AA genotype is significantly superior to the GG genotype in frontal width. Conversely, the GG genotype surpasses the AA genotype in various other traits. In particular, the GG genotype significantly outperforms the AG genotype in body height and exhibits very significant superiority over the AG genotype in tube circumference. For chest depth, the GG genotype is significantly superior to the AA genotype. At site C413217T, the CC genotype excels the CT genotype in several body size metrics, including weight, body height, back height, waist height, sacral height, limb length, straight body length, frontal width, and tube circumference. Chest circumference CC genotypes were significantly better than CT genotypes.

**Table 7:** Body size performance of the C413217T and A431965G sites in Australian White × Small-tailed Han Sheep crossbred generation ewes.

Name	Australian White × Small-tailed Han Sheep crossbred generation ewes				
Site	A431965G	A431965G	A431965G	C413217T	C413217T
Genotype	AA (64/120)	AG (16/120)	GG (40/120)	CC (116/120)	CT (4/120)
Weight (kg)	60.41 ± 1.27	58.00 ± 1.74	60.35 ± 1.61	60.36 ± 0.91	51.50 ± 0.00
Body height (cm)	72.34 ± 0.44 ${}^{\mathrm{ab}}$	71.25 ± 0.34 ${}^{b}$	75.43 ± 0.62 ${}^{a}$	73.33 ± 0.36	70.00 ± 0.00
Back height (cm)	72.49 ± 0.41	71.63 ± 0.31	74.85 ± 0.54	73.28 ± 0.31	69.80 ± 0.00
Waist height (cm)	73.91 ± 0.46	73.50 ± 0.53	76.05 ± 0.67	74.67 ± 0.36	71.50 ± 0.00
Sacral height (cm)	74.77 ± 0.40	75.13 ± 0.49	76.34 ± 0.69	75.42 ± 0.34	72.80 ± 0.00
Hip height (cm)	65.63 ± 0.56	69.83 ± 1.49	69.11 ± 0.70	67.29 ± 0.47	69.00 ± 0.00
Frontal width (cm)	16.03 ± 0.13 ${}^{a}$	15.13 ± 0.06 ${}^{\mathrm{ab}}$	14.84 ± 0.11 ${}^{b}$	15.57 ± 0.09 ${}^{a}$	14.00 ± 0.00 ${}^{b}$
Tube circumference (cm)	9.49 ± 0.08 ${}^{\mathrm{bAB}}$	8.70 ± 0.15 ${}^{\mathrm{bB}}$	9.62 ± 0.07 ${}^{\mathrm{aA}}$	9.46 ± 0.06 ${}^{a}$	8.50 ± 0.00 ${}^{b}$
Chest circumference (cm)	109.47 ± 1.07	110.00 ± 1.20	110.70 ± 1.34	110.33 ± 0.74 ${}^{a}$	99.00 ± 0.00 ${}^{b}$
Limb length (cm)	21.69 ± 0.19	21.63 ± 0.48	22.75 ± 0.26	22.10 ± 0.16	20.00 ± 0.00
Leg length (cm)	51.38 ± 0.64	49.75 ± 0.90	50.60 ± 0.95	50.78 ± 0.49	54.50 ± 0.00
Straight body length (cm)	61.88 ± 0.59	60.38 ± 1.02	65.05 ± 0.82	62.83 ± 0.48	60.00 ± 0.00
Chest depth (cm)	35.77 ± 0.26 ${}^{b}$	37.70 ± 0.50 ${}^{\mathrm{ab}}$	38.76 ± 0.42 ${}^{a}$	36.70 ± 0.25	37.80 ± 0.00
Chest width (cm)	29.01 ± 0.40	28.58 ± 0.54	29.37 ± 0.67	29.01 ± 0.32	31.00 ± 0.00
Waist angle width (cm)	23.48 ± 0.54	23.95 ± 1.12	25.74 ± 0.37	24.24 ± 0.37	26.00 ± 0.00
Hip width (cm)	28.70 ± 0.55	30.20 ± 0.84	28.09 ± 0.83	28.58 ± 0.43	32.00 ± 0.00

Note: different lower-cased letter superscripts indicate significant differences at the 0.05 level, while upper-cased letter superscripts indicate differences at the 0.01 level.

Table 8 presents insights into the impact of two SNP mutation sites on body size performance in Australian White ewes. At site A431965G, the AA genotype demonstrates superiority over the GG genotype in specific traits, including the weight, frontal width, limb length, leg length, body straight length, waist angle width, and AG genotype being superior to the AA genotype for the other traits.

**Table 8:** Body size performance of the C413217T and A431965G sites in Australian White ewes.

Name	Australian White ewes		
Site	A431965G	A431965G	C413217T
Genotype	AA (104/120)	AG (16/120)	CC (120/120)
Weight (kg)	64.85 ± 1.06	62.38 ± 1.86	64.52 ± 0.96
Body height (cm)	70.00 ± 0.31	71.75 ± 1.39	70.23 ± 0.33
Back height (cm)	69.36 ± 0.31	70.50 ± 1.30	69.51 ± 0.32
Waist height (cm)	70.27 ± 0.33	72.38 ± 0.89	70.55 ± 0.31
Sacral height (cm)	70.31 ± 0.29	72.00 ± 1.25	70.53 ± 0.30
Hip height (cm)	63.62 ± 0.56	64.50 ± 1.53	63.73 ± 0.53
Frontal width (cm)	14.87 ± 0.08	14.38 ± 0.11	14.80 ± 0.07
Tube circumference (cm)	9.00 ± 0.05	9.25 ± 0.06	9.03 ± 0.04
Chest circumference (cm)	109.92 ± 0.82	112.63 ± 1.12	110.28 ± 0.73
Limb length (cm)	21.19 ± 0.14	21.00 ± 0.48	21.17 ± 0.13
Leg length (cm)	47.02 ± 0.43	43.63 ± 0.68	46.56 ± 0.40
Straight body length (cm)	63.73 ± 0.42	63.50 ± 1.61	63.70 ± 0.41
Chest depth (cm)	35.87 ± 0.28	36.18 ± 0.89	35.91 ± 0.27
Chest width (cm)	30.41 ± 0.34	31.23 ± 1.22	30.52 ± 0.33
Waist angle width (cm)	26.72 ± 0.50	25.15 ± 0.74	26.51 ± 0.45
Hip width (cm)	25.93 ± 0.36	25.45 ± 0.51	25.86 ± 0.32

### 3.6 Haplotype combinations of the two SNP sites

The analysis of the two SNP sites using SHEsis (http://analysis.bio-x.cn/myAnalysis.php, last access: 23 May 2023) revealed the potential formations of nine haplotype combinations. However, it is noteworthy that only five haplotype combinations were identified across all the sheep, as illustrated in Table 9.

**Table 9:** Haplotype combinations of the two SNP sites.

Haplotype	H1: CC	H2: CT	H3: TT
H1: AA	AACC (697/1137)	AACT (279/1137)	AATT (29/1137)
H2: AG	AGCC (52/1137)	AGCT	AGTT
H2: GG	GGCC (80/1137)	GGCT	GGTT

### 3.7 Association analysis of haplotype combinations with reproductive performance

Table 10 provides a clear overview of the haplotypes identified in the four sheep breeds, i.e., AACC, AACT, AATT, AGCC, and GGCC. Notably, the AACT haplotype stands out with higher lambing numbers compared to the other haplotypes. Furthermore, it is worth highlighting that Charolais sheep exhibited significantly higher lambing numbers in comparison to the AATT and GGCC haplotypes.

**Table 10:** Number of lambs produced by haplotype combinations in various breeds of sheep.

Name	Haplotype	Number	Number of lambs
Charolais ewes	AACC	497	1.76 ± 0.02 ${}^{A}$
	AACT	239	1.77 ± 0.03 ${}^{A}$
	AATT	29	1.00 ± 0.00 ${}^{B}$
	GGCC	12	1.00 ± 0.00 ${}^{B}$
Charolais × Small-tailed Han Sheep crossbred generation ewes	AACC	36	2.00 ± 0.00
	AACT	36	2.00 ± 0.00
	AGCC	20	2.00 ± 0.00
	GGCC	28	1.00 ± 0.00
Australian White × Small-tailed Han Sheep crossbred generation ewes	AACC	60	2.00 ± 0.00
	AACT	4	2.00 ± 0.00
	AGCC	16	2.00 ± 0.00
	GGCC	40	1.00 ± 0.00
Australian White ewes	AACC	104	2.00 ± 0.00
	AGCC	16	2.00 ± 0.00

Note: different lower-cased letter superscripts indicate significant differences at the 0.05 level, while upper-cased letter superscripts indicate differences at the 0.01 level.

### 3.8 Association analysis of haplotype combinations with body size performance

Within the Charolais ewes, four distinct haplotypes were identified. Notably, body height exhibited a significant increase within the GGCC haplotype compared to the AATT haplotype. Moreover, tube circumference was notably higher in the AACC haplotype, significantly surpassing the measurements observed in the GGCC haplotype. Additionally, both chest depth and width were found to be significantly more favorable in the AACT haplotype when compared to the AATT haplotype. Consequently, it can be concluded that the AACT haplotype emerges as the dominant genotype within this context, as depicted in Table 11.

**Table 11:** Body size performance of haplotype combinations in Charolais ewes.

Name	Charolais ewes			
Haplotype	AACC (497/777)	AACT (239/777)	AATT (29/777)	GGCC (12/777)
Weight (kg)	79.60 ± 0.81	79.14 ± 1.45	69.00 ± 0.00	70.83 ± 1.48
Body height (cm)	71.79 ± 0.20 ${}^{\mathrm{abAB}}$	71.24 ± 0.32 ${}^{\mathrm{abAB}}$	68.00 ± 0.00 ${}^{\mathrm{bB}}$	73.33 ± 1.24 ${}^{\mathrm{aA}}$
Back height (cm)	71.41 ± 0.21	70.76 ± 0.30	70.00 ± 0.00	71.67 ± 0.86
Waist height (cm)	71.80 ± 0.24	71.53 ± 0.28	72.00 ± 0.00	71.50 ± 0.98
Sacral height (cm)	71.13 ± 0.28	71.42 ± 0.20	72.00 ± 0.00	72.67 ± 0.86
Hip height (cm)	64.12 ± 0.39	64.31 ± 0.47	63.00 ± 0.00	68.33 ± 0.86
Frontal width (cm)	15.58 ± 0.06	15.32 ± 0.08	15.00 ± 0.00	16.00 ± 0.21
Tube circumference (cm)	9.89 ± 0.05 ${}^{\mathrm{aA}}$	9.40 ± 0.08 ${}^{\mathrm{abAB}}$	9.00 ± 0.00 ${}^{\mathrm{bcAB}}$	8.50 ± 0.00 ${}^{\mathrm{cB}}$
Chest circumference (cm)	125.45 ± 0.66	126.05 ± 1.00	130.00 ± 0.00	122.67 ± 1.53
Limb length (cm)	21.65 ± 0.17	20.98 ± 0.20	22.00 ± 0.00	23.00 ± 0.42
Leg length (cm)	47.80 ± 0.43	46.00 ± 0.79	48.00 ± 0.00	40.83 ± 1.20
Straight body length (cm)	64.36 ± 0.29	63.31 ± 0.68	67.00 ± 0.00	63.00 ± 1.54
Chest depth (cm)	36.28 ± 0.18 ${}^{a}$	36.86 ± 0.20 ${}^{a}$	32.90 ± 0.00 ${}^{b}$	36.37 ± 0.75 ${}^{a}$
Chest width (cm)	34.53 ± 0.22 ${}^{\mathrm{ab}}$	35.92 ± 0.29 ${}^{a}$	31.00 ± 0.00 ${}^{b}$	33.03 ± 0.49 ${}^{\mathrm{ab}}$
Waist angle width (cm)	31.35 ± 0.39	33.59 ± 0.49	33.20 ± 0.00	33.43 ± 0.80
Hip width (cm)	34.40 ± 0.23	34.60 ± 0.35	31.00 ± 0.00	31.70 ± 1.10

Note: different lower-cased letter superscripts indicate significant differences at the 0.05 level, while upper-cased letter superscripts indicate differences at the 0.01 level.

In the crossbred generation of Charolais × Small-tailed Han Sheep ewes, the analysis revealed the presence of four distinct haplotypes. Notably, the AACC haplotype exhibited a significantly greater weight compared to the other three haplotypes. Furthermore, the AACC haplotype also showed significant advantages over the AGCC haplotype in terms of body height and breast circumference. Most notably, the AACC haplotype significantly outperformed the AGCC haplotype in loin angle width. These findings collectively indicate that the AACC haplotype emerges as the dominant haplotype in this context, as summarized in Table 12.

**Table 12:** Body size performance of haplotype combinations in Charolais × Small-tailed Han Sheep crossbred generation ewes.

Name	Charolais × Small-tailed Han Sheep crossbred generation ewes			
Haplotype	AACC (36/120)	AACT (36/120)	AGCC (20/120)	GGCC (28/120)
Weight (kg)	77.90 ± 2.00 ${}^{a}$	61.83 ± 1.73 ${}^{b}$	62.20 ± 0.24 ${}^{b}$	62.86 ± 2.28 ${}^{b}$
Body height (cm)	78.71 ± 0.70 ${}^{a}$	75.50 ± 0.76 ${}^{\mathrm{ab}}$	71.90 ± 0.40 ${}^{b}$	76.50 ± 0.69 ${}^{\mathrm{ab}}$
Back height (cm)	78.55 ± 0.76	75.67 ± 0.68	73.70 ± 0.20	76.07 ± 0.64
Waist height (cm)	79.45 ± 0.83 ${}^{a}$	76.78 ± 0.62 ${}^{\mathrm{ab}}$	73.90 ± 0.18 ${}^{b}$	75.54 ± 0.59 ${}^{\mathrm{ab}}$
Sacral height (cm)	78.27 ± 0.70	76.39 ± 0.68	75.00 ± 0.46	73.36 ± 1.31
Hip height (cm)	68.40 ± 0.48	68.22 ± 0.93	67.00 ± 0.52	66.57 ± 1.36
Frontal width (cm)	15.50 ± 0.21	15.67 ± 0.16	15.50 ± 0.23	15.00 ± 0.16
Tube circumference (cm)	10.10 ± 0.19	9.94 ± 0.17	9.60 ± 0.27	9.64 ± 0.16
Chest circumference (cm)	131.85 ± 1.68 ${}^{a}$	123.61 ± 1.38 ${}^{\mathrm{ab}}$	120.40 ± 1.43 ${}^{b}$	122.21 ± 1.34 ${}^{\mathrm{ab}}$
Limb length (cm)	21.80 ± 0.29	22.11 ± 0.36	21.40 ± 0.23	21.57 ± 0.26
Leg length (cm)	51.30 ± 1.11	52.56 ± 1.07	50.40 ± 0.94	56.64 ± 0.41
Straight body length (cm)	66.35 ± 0.87	66.94 ± 1.26	63.00 ± 1.56	60.14 ± 0.65
Chest depth (cm)	40.72 ± 0.59	37.62 ± 0.52	38.72 ± 0.65	39.50 ± 1.06
Chest width (cm)	35.39 ± 0.82	33.28 ± 0.54	31.40 ± 1.10	31.79 ± 0.63
Waist angle width (cm)	30.90 ± 0.59 ${}^{\mathrm{aA}}$	26.70 ± 0.65 ${}^{\mathrm{abAB}}$	23.76 ± 0.68 ${}^{\mathrm{bB}}$	27.74 ± 0.63 ${}^{\mathrm{abAB}}$
Hip width (cm)	34.50 ± 1.07	31.64 ± 0.92	32.56 ± 1.29	31.93 ± 0.89

Note: different lower-cased letter superscripts indicate significant differences at the 0.05 level, while upper-cased letter superscripts indicate differences at the 0.01 level.

In the crossbred generation of Australian White × Small-tailed Han Sheep ewes, the analysis identified four distinct haplotypes. Notably, the GGCC haplotype exhibited a significant advantage over the AACT haplotype in terms of body height. Additionally, the AACC haplotype was highly significantly superior to the AACT haplotype in frontal width, while the GGCC haplotype was very significantly higher than the AACT haplotype in both tube circumference and breast circumference. These findings collectively indicate that the GGCC haplotype stands out as the dominant genotype within this context, as summarized in Table 13.

**Table 13:** Body size performance of haplotype combinations in Australian White × Small-tailed Han Sheep crossbred generation ewes.

Name	Australian White × Small-tailed Han Sheep crossbred generation ewes			
Haplotype	AACC (60/120)	AACT (4/120)	AGCC (16/120)	GGCC (40/120)
Weight (kg)	61.00 ± 1.32	51.50 ± 0.00	58.00 ± 1.74	60.35 ± 1.62
Body height (cm)	72.50 ± 0.47 ${}^{\mathrm{ab}}$	70.00 ± 0.00 ${}^{b}$	71.25 ± 0.34 ${}^{\mathrm{ab}}$	75.43 ± 0.62 ${}^{a}$
Back height (cm)	72.67 ± 0.43 ${}^{\mathrm{ab}}$	69.80 ± 0.00 ${}^{b}$	71.63 ± 0.31 ${}^{\mathrm{ab}}$	74.85 ± 0.54 ${}^{a}$
Waist height (cm)	74.07 ± 0.49	71.50 ± 0.00	73.50 ± 0.53	76.05 ± 0.67
Sacral height (cm)	74.90 ± 0.43	72.80 ± 0.00	75.13 ± 0.49	76.34 ± 0.69
Hip height (cm)	65.40 ± 0.58	69.00 ± 0.00	69.83 ± 1.48	69.11 ± 0.70
Frontal width (cm)	16.17 ± 0.12 ${}^{\mathrm{aA}}$	14.00 ± 0.00 ${}^{\mathrm{cB}}$	15.13 ± 0.05 ${}^{\mathrm{bAB}}$	14.84 ± 0.11 ${}^{\mathrm{bcAB}}$
Tube circumference (cm)	9.55 ± 0.08 ${}^{\mathrm{aA}}$	8.50 ± 0.00 ${}^{\mathrm{bB}}$	8.70 ± 0.15 ${}^{\mathrm{bAB}}$	9.62 ± 0.07 ${}^{\mathrm{aA}}$
Chest circumference (cm)	110.17 ± 1.08 ${}^{a}$	99.00 ± 0.00 ${}^{b}$	110.00 ± 1.20 ${}^{a}$	110.70 ± 1.34 ${}^{a}$
Limb length (cm)	21.80 ± 0.20 ${}^{\mathrm{ab}}$	20.00 ± 0.00 ${}^{b}$	21.63 ± 0.48 ${}^{\mathrm{ab}}$	22.75 ± 0.26 ${}^{a}$
Leg length (cm)	51.17 ± 0.67	54.50 ± 0.00	49.75 ± 0.90	50.60 ± 0.95
Straight body length (cm)	62.00 ± 0.62	60.00 ± 0.00	60.38 ± 1.02	65.05 ± 0.82
Chest depth (cm)	35.63 ± 0.27	37.80 ± 0.00	37.70 ± 0.50	38.76 ± 0.42
Chest width (cm)	28.89 ± 0.42	31.00 ± 0.00	28.58 ± 0.54	29.37 ± 0.67
Waist angle width (cm)	23.31 ± 0.56	26.00 ± 0.00	23.95 ± 1.11	25.74 ± 0.36
Hip width (cm)	28.48 ± 0.58	32.00 ± 0.00	30.20 ± 0.84	28.09 ± 0.83

Note: different lower-cased letter superscripts indicate significant differences at the 0.05 level, while upper-cased letter superscripts indicate differences at the 0.01 level.

In the crossbred generation of Australian White ewes, two distinct haplotypes were identified. Notably, the AACC haplotype exhibited superiority over the AGCC haplotype in several key characteristics, including body weight, frontal width, limb length, leg length, straight body length, and waist angle width. Conversely, the AGCC haplotype outperformed the AACC haplotype in other traits. Based on these findings, it is evident that the AACC haplotype emerges as the dominant haplotype within this context, as demonstrated in Table 14.

**Table 14:** Body size performance of haplotype combinations in Australian White ewes.

Name	Australian White ewes	
Haplotype	AACC (104/120)	AGCC (16/120)
Weight (kg)	64.85 ± 1.06	62.38 ± 1.86
Body height (cm)	70.00 ± 0.31	71.75 ± 1.39
Back height (cm)	69.36 ± 0.32	70.50 ± 1.30
Waist height (cm)	70.27 ± 0.33	72.38 ± 0.89
Sacral height (cm)	70.30 ± 0.29	72.00 ± 1.25
Hip height (cm)	63.62 ± 0.56	64.50 ± 1.53
Frontal width (cm)	14.87 ± 0.07	14.38 ± 0.11
Tube circumference (cm)	9.00 ± 0.05	9.25 ± 0.06
Chest circumference (cm)	109.92 ± 0.82	112.62 ± 1.12
Limb length (cm)	21.19 ± 0.14	21.00 ± 0.48
Leg length (cm)	47.01 ± 0.43	43.63 ± 0.68
Straight body length (cm)	63.73 ± 0.41	63.50 ± 1.16
Chest depth (cm)	35.87 ± 0.28	36.18 ± 0.89
Chest width (cm)	30.41 ± 0.34	31.22 ± 1.22
Waist angle width (cm)	26.72 ± 0.50	25.15 ± 0.74
Hip width (cm)	25.93 ± 0.36	25.45 ± 0.51

## 4 Discussion

Reproductive traits have significant economic importance in the context of sheep farming. The selection of sheep breeds with high reproductive yields plays a pivotal role in the industry's development. Among the genes associated with reproductive traits in sheep, the *FecB* genes stand out as extensively studied, with their mechanisms of action well understood. Presently, *FecB* mutations have been identified in various sheep breeds, including Booroola (Mulsant et al., 2001), Small-tailed Han Sheep (Davis et al., 2006; Liu et al., 2003), Garole (Davis et al., 2002), and Hu sheep (Guan et al., 2007). In our study, mutations in the *FecB* gene were identified in four different breeds of sheep, and these mutations were found to impact reproductive performance. It is worth noting that the frequencies of these alleles carrying the *FecB* mutation vary across different breeds. Recent years have seen a surge in research exploring the impact of ewes carrying the *FecB* gene in their offspring. However, the consensus among most researchers suggests that the *FecB* gene tends to have a negative effect on the body size and weight of the resulting lambs. For instance, in the study conducted by Cui (2010), the influence of the *FecB* gene on the growth and development of Dorper sheep × Small-tailed Han Sheep crossbred meat lambs was examined. The findings revealed that B ${}^{+}$ -type lambs exhibited smaller birth weights, body measurements, and 3-month-old weaning weights compared to the ${}^{++}$ -type lambs (Cui et al., 2010). These differences were particularly pronounced in the case of ewe lambs. In another study by Sejian on Garole × Malpura crossbred sheep, it was observed that the presence of the *FecB* gene led to a significant reduction in body weight, chest circumference, body width, and body length in ewes. However, paradoxically, it resulted in lambs with higher birth weights compared to ewes without the *FecB* gene (Sejian et al., 2015). Similarly, Oraon's investigation of Chhotanagpuri sheep in India demonstrated that BB- and B ${}^{+}$ -type lambs weighed significantly less than ${}^{++}$ -type lambs at 52 weeks of age (Oraon et al., 2016). Furthermore, Zhang reported that the *FecB* mutation had an adverse impact on the birth weight and body measurements of lambs, with ${}^{++}$ types exhibiting significantly higher values compared to B ${}^{+}$ types (Zhang et al., 2021). However, it is worth noting that there was no significant effect on the weight and body size of lambs at 3 months of age. In the aforementioned studies, the emphasis has been predominantly on the impact on lambs, with limited reporting on the influence of maternal body size traits. However, some research has indicated that maternal body size characteristics can indeed have effects on lamb (Näsholm and Danell, 1996). Therefore, we investigated ewes from four different breeds, conducting not only the detection of mutations in the *FecB* gene, but also correlational analyses to explore the impact of these mutations on their reproductive and body size traits. This effort aims to address the existing gap in this aspect of research.

The *FecB* gene holds paramount significance in enhancing the reproductive performance of sheep and is widely employed to improve the reproductive outcomes within sheep populations. In recent years, researchers have consistently explored various approaches, including hybridization, single-base editing techniques, and transgenic technologies, to introduce the *FecB* gene into less fertile sheep populations. In one notable study conducted by Zhang et al. (2021), white-headed Dorper sheep were utilized as the male parent, while Hu sheep and Duhu hybrid ewes were selected as the female parent for hybridization. The outcomes of this research indicated that the introduction of the *FecB* gene had a discernible impact on the lambing rates in both the first and second generations of Duhu ewes. Furthermore, Luo implemented a breeding strategy by crossing male Hu sheep carrying the BB type of the *FecB* gene with local Kazakh ewes. This endeavour led to the initial cultivation of a new type of multiple birth among Kazakh sheep, showcasing the potential for enhancing reproductive traits (Luo, 2017). In another innovative approach, Zhou harnessed adenine base editor (ABE) technology to obtain sheep with the *FecB* gene (Zhou et al., 2020). Additionally, Yu et al. (2012) successfully extracted the *FecB* gene from Hu sheep and introduced it into Xinjiang fine-wool sheep through transgenic technology. This ground-breaking achievement laid a solid foundation for the development of Xinjiang fine-wool sheep with a significantly improved reproduction rate.

The focus of the current study was to investigate the influence of different genotypes at various sites of the *FecB* gene and the resulting haplotype combinations on the reproductive and body size performance of diverse sheep breeds. Our findings revealed several key insights. Firstly, we observed variations in gene frequency and genotype frequency at the same SNP site across different sheep breeds, which is consistent with the findings of Hua and Yang (2009). No studies had been conducted on these four breeds before our research, and our study serves to fill this gap in the existing literature. Moreover, differences in gene frequency and genotype frequency were also evident at different SNP sites within the same breed of sheep. We speculate that the emergence of this phenomenon may be attributed to the exertion of intense artificial selection. Notably, the C413217T site exhibited moderate polymorphism in Charolais ewes, while the A431965G site displayed similar characteristics in Charolais × Small-tailed Han Sheep crossbred generation ewes and Australian White × Small-tailed Han Sheep crossbred generation ewes. These observations suggest the potential for optimizing production performance associated with these sites. Furthermore, the higher heterozygosity observed at the A431965G site in Charolais × Small-tailed Han Sheep crossbred generation ewes and Australian White × Small-tailed Han Sheep crossbred generation ewes indicates a richer genetic resource and greater genetic diversity in these populations. Regarding genotypes and reproductive performance, the CT genotype at the C413217T site displayed significant superiority over the TT genotype. Similarly, the AA genotype at the A431965G site was correlated with a higher number of lambs produced compared to the GG genotype, indicating that the CT and AA genotypes are likely to be dominant. In the context of haplotype combinations, AACT emerged as significantly superior to GGCC and AATT, establishing AACT as the dominant haplotype. For analyses of genotypes and body performance, the AA type outperformed the AG and GG types in weight, chest circumference, and straight body length in Charolais and Australian White × Small-tailed Han Sheep crossbred generation ewes at the A431965G site. This is consistent with the findings of Sejian et al. (2015). The superiority of the AA genotype over other genotypes, except for the Australian White × Small-tailed Han Sheep crossbred generation ewes, at the hip width indicates potential differences between breeds. Additionally, at the C413217T locus, the CC genotype generally outperforms the CT and TT genotypes in most body size traits, while the CT genotype excels in hip width over other genotypes. The width of the ewe's hips may indeed have an impact on lambing, as it is related to the structure of the reproductive organs and the width of the birth canal. The dominant haplotype combinations formed by hip width align with those observed for reproductive traits, confirming the dominance of certain haplotypes in reproductive performance. In terms of most body size traits, the AACC haplotype is identified as the dominant haplotype. Furthermore, the CC genotype was superior to the CT and TT genotypes at the C413217T site, with AACC emerging as the dominant haplotype. It is essential to note that haplotype combinations often yield more convincing results than individual genotypes. The effects of haplotype combinations are not merely additive but interact with each other. As such, haplotype combinations provide a more comprehensive and effective means of evaluating production traits or population genetic improvement. Breeders can utilize this information to intervene in haplotype frequencies within populations to achieve specific breeding goals tailored to their production needs.

## 5 Conclusions

In this study, two SNP sites in the *FecB* gene were detected in Charolais ewes, Australian White ewes, Charolais × Small-tailed Han Sheep crossbred generation ewes, and Australian White × Small-tailed Han Sheep crossbred generation ewes. The results showed that the dominant genotypes for reproductive performance were the AA and CT genotypes, with the dominant haplotype combination of AACT, and the dominant genotypes for somatic performance were the AA and CC genotypes, with the dominant haplotype combination of AACC. These advantageous genotypes and haplotype combinations hold considerable promise for the selection and enhancement of sheep breeds. They represent a valuable resource for advancing the field of sheep genetic breeding and play a pivotal role in providing the essential theoretical underpinnings necessary for the development of higher-fertility sheep.

## Data Availability

The data sets used in this article can be requested from the corresponding author.
